# Supplementary material for: Understanding the Multidimensional Effects of Polymorphism, Particle Size and Processing for D-Mannitol Powders
Source: Pharmaceutics. 2022 Oct 7;14(10):2128. doi: 10.3390/pharmaceutics14102128 (PMC9611586; doi:10.3390/pharmaceutics14102128)
Supplement: Supplementary file 1 [file pharmaceutics-14-02128-s001.zip › pharmaceutics-1955984-supplementary.pdf]

pharmaceutics

MDPI

Supplement

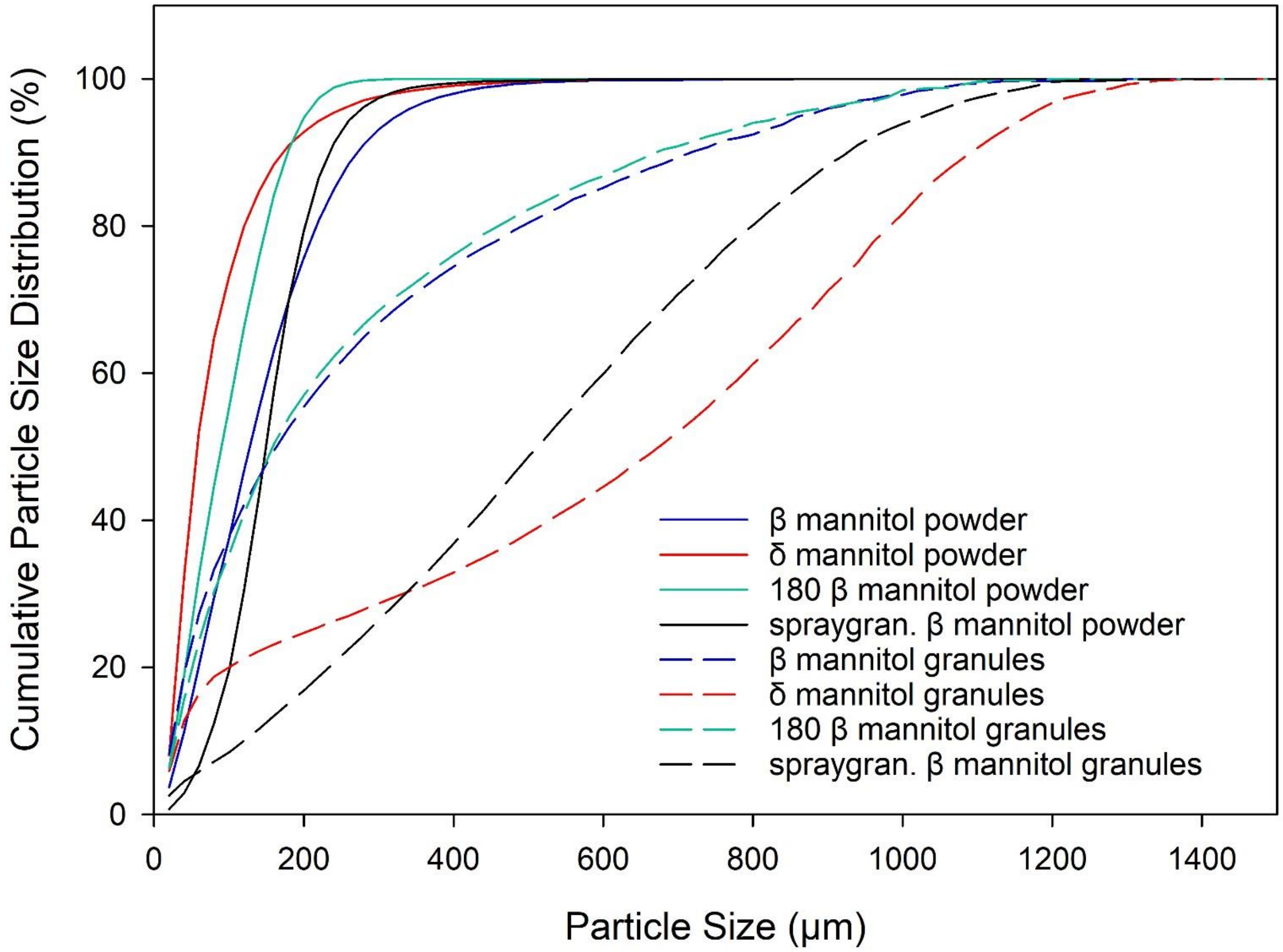

Figure S1. Cumulative particle size distribution curves of δ mannitol, β mannitol, β mannitol sieved through 180 µm sieve, spray granulated β mannitol and their respective granules. Arithmetic means of n=3.

Table S1. Pycnometric density of δ and β mannitol, β mannitol sieved through 180 µm sieve and spray granulated β mannitol powder and their respective granules. Arithmetic means of n=3 ± S.D.

	δ mannitol density (g/mL)	β mannitol density (g/mL)	180 β mannitol density (g/mL)	spray granulated β mannitol density (g/mL)
powder	1.5098 ± 0.0003	1.4975 ± 0.0003	1.4976 ± 0.0005	1.5105 ± 0.0027
granules	1.5102 ± 0.0003	1.5012 ± 0.0001	1.4968 ± 0.0008	1.5130 ± 0.0029

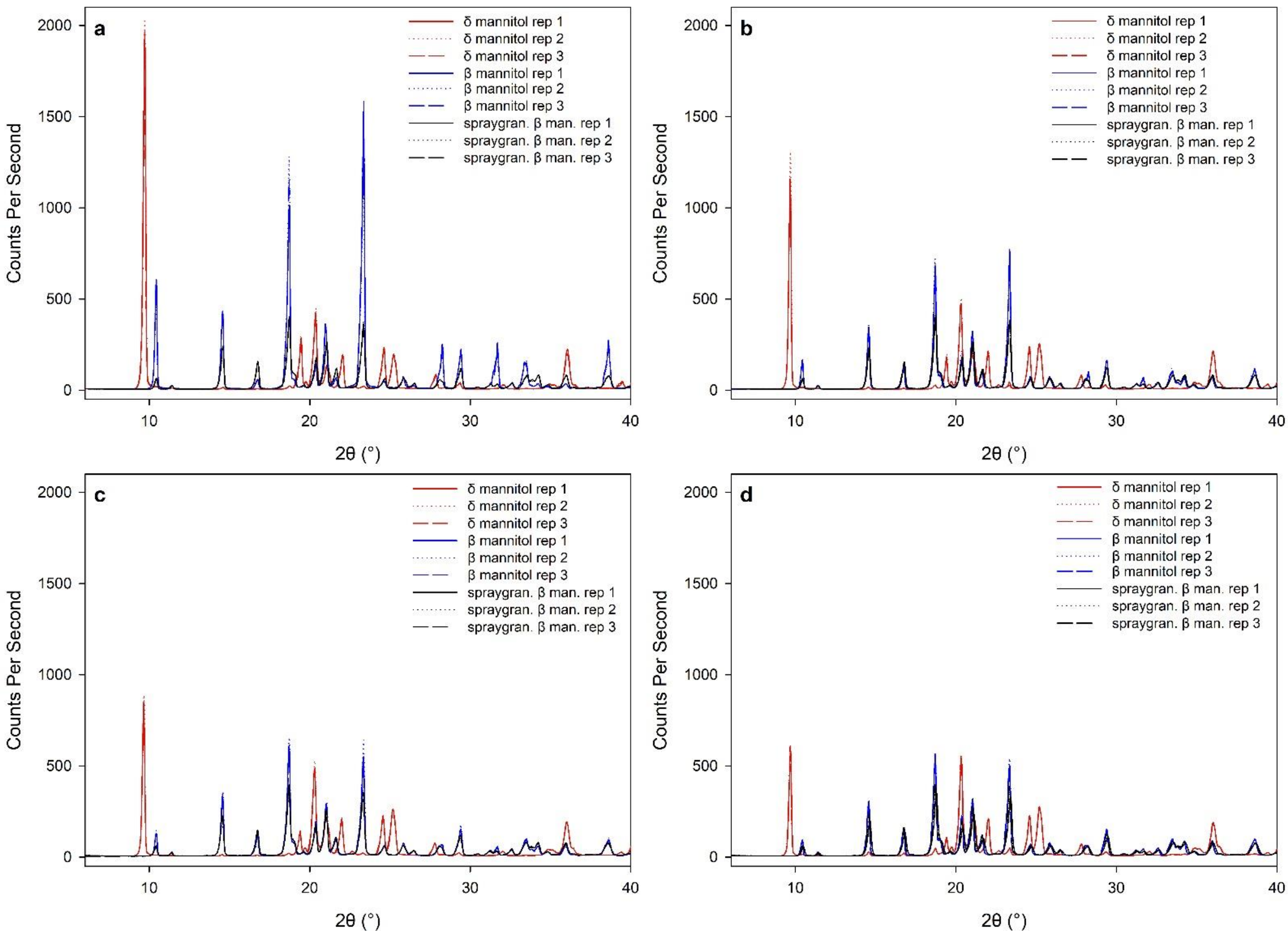

Figure S2. X-ray powder diffractograms of δ mannitol, β mannitol and spray granulated β mannitol (a) powder, (b) granules, (c) tablets produced from powder, and (d) tablets produced from the granules.

A Bruker D2 Phaser (Bruker Corporation, USA) equipped with a SSD160 detector was used for polymorph characterization via Powder X-ray diffractometry (PXRD). Measurements were performed in reflection geometry with a Cu-Kα1 radiation source at 30kV and 10 mA. A 2θ range of 6° 2θ to 41° 2θ with measurement times of 1 sec / PSD-step and a PSD step width of 0.02° 2θ was applied. Tablets were mortared for 3 minutes before measurement and 3 replicates for powder, granules and respective tablets for each mannitol were measured.
